# Integrating multivariate analysis and Air Pollution Tolerance Index (APTI) to evaluate four ornamental plants for sustainable indoor air phytoremediation

**DOI:** 10.1038/s41598-026-50763-0

**Published:** 2026-05-23

**Authors:** Safinaz M. Elhadad, Shalaby ea, Ibrahim H. Saleh, Mohamed Y. Omar

**Affiliations:** 1 College of pharmacy, Arab Academy for Science, Technology and Maritime Transport, Alexandria, Egypt; 2 Department of environmental studies, Institute of Graduate Studies and Research, Alexandria, Egypt; 3 Deanery of Scientific Research & Innovation, Arab Academy for Science, Technology and Maritime Transport, Alexandria, Egypt

**Keywords:** Air phytoremediation, Indoor air pollution, Multivariate analysis, Pharmaceutical laboratory, Volatile organic compounds (VOCs), Environmental sciences, Plant sciences

## Abstract

Indoor air pollution, especially in pharmaceutical laboratories, poses significant health risks due to the presence of volatile organic compounds (VOCs) such as benzene, toluene, acetophenone, and benzaldehyde. This study evaluates the efficiency of air phytoremediation technology using four ornamental plant species, *Cordyline fruticosa*, *Syngonium podophyllum*, *Epipremnum aureum* and *Chlorophytum comosum* to improve Indoor Air Quality (IAQ) by acting as Plant-Based Bio-Filters (PBBFs) in both pot-based and green wall configurations. VOC concentrations were monitored in a real pharmaceutical organic laboratory. Morphological and physiological plant traits including total chlorophyll content, relative water content (RWC), leaf pH, ascorbic acid concentration, stomatal density, and cuticle wax content were evaluated. Air Pollution Tolerance Index (APTI) and dust-capturing potential were calculated to assess the resilience and effectiveness of each species under VOCs exposure. Chemometric tools Principal Component Analysis (PCA) and Orthogonal Projections to Latent Structures-Discriminant Analysis (OPLS-DA) were applied to identify species with superior removal efficiency and to explore the relationship between plant traits and VOC uptake. Among the studied species, *Cordyline fruticosa* demonstrated the highest removal efficiency for VOCs (87.50%), CO (88.23%), and CO₂ (36.78%), as well as the highest APTI (14.76%), stomatal density (94.34 stomata/mm^2^), and chlorophyll content. *Syngonium podophyllum* also showed up to 100% removal of particulate matter (PM_2.5_ and PM_10_) and performed effectively in CO (70.58%) and CO₂ (31.27%) reduction. Multivariate analysis confirmed that plants with higher physiological resilience and morphological surface complexity had significantly greater phytoremediation capacity. This study demonstrate the potential of PBBFs, especially using *Cordyline fruticosa* and *Syngonium podophyllum*, as a viable, cost-effective, and sustainable approach to mitigate indoor VOCs and improve air quality in pharmaceutical labs. The findings support integrating ornamental plants into indoor environment as a natural solution for IAQ management.

## Introduction

Volatile Organic Compounds (VOCs) are key contributors to poor indoor air quality (IAQ), especially in pharmaceutical laboratories. Prolonged exposure to VOCs such as benzene, toluene, acetophenone, and benzaldehyde poses significant health risks^1^. Botanical biofilters or plant-based biofilters (PBBFs) offer a passive, sustainable method to reduce airborne pollutants^2^. Previous research has highlighted the role of ornamental plants in VOC absorption, yet limited studies address their real-world application in pharmaceutical labs^3^. In addition to VOC uptake through plant roots and leaves, several morphological and physiological traits play critical roles in determining phytoremediation efficiency. The Air Pollution Tolerance Index (APTI), which integrates total chlorophyll content, ascorbic acid level, relative water content (RWC), and leaf extract pH, serves as a robust indicator of a plant’s resistance to air pollutants. High APTI values suggest strong stress tolerance and higher pollutant absorption capacity^4^..

A few plants have the capacity to absorb, corrupt, or adjust harmful poisons through phytovolatilization is frequently considered advantageous. The kind of plant activity being employed in these techniques depends on the nature of the compounds being remediated and the type of plant parts being used in the process^4^. Air pollutant VOCs can be effectively removed by plants. For example, *Brassica* species absorb SO_2_ and NO_2_ from polluted air, *Chenopodium murale* removes volatile hydrocarbons, *Zamioculcas zamiifolia* removes formaldehyde^5^,Trichloroethylene (TCE) and tetrachloroethylene (PCE) removed by *Salix* species^6^. The bio-concentration levels of total VOCs in *Epipremnum aureum* were noted as 74.71 to 174.42, signifying that *E. aureum* is effective for removal of VOCs naturally and sustainably. The tropical *leguminous* tree *Leucaena leucocephala* was studied to treat pharmaceutical contaminants such as ethylene dibromide and trichloroethylene^7^.

To successfully remove air pollutants, several research have many aspects of plant properties, including leaf area, number, sunlight intensity, respiration, and photosynthesis (Irga, 2017) estimated that 57 m^2^ of leaf area would be able to absorb/remove around 13% of CO_2_ generated per person in an average room without ventilation. Other leaf properties, including thickness, roughness, and villi, were also significant factors in relation to dust retention capacity^8^. Dust exposure causes physiological changes in leaves, leading to visible damage symptoms. Tolerance to air pollution is conferred through physiological and biochemical adjustments, such as pH, relative water content, total chlorophyll, and ascorbic acid. These parameters are used to calculate the air pollution tolerance index (APTI) in plants. Sensitive species can act as biological indicators or biomonitors for air quality, and tolerant species can be used as sinks in green belt development plans. Most studies on APTI are related to industrial activities and vehicles, but few reports on natural dust.^9^. Furthermore, stomatal density and distribution facilitate gaseous pollutant intake through leaf pores, significantly influencing VOC uptake rates. Plants with higher stomatal density and openness allow for increased air pollutant exchange and removal. Likewise, the presence of cuticle wax on the leaf surface enhances the adsorption of VOCs and particulate matter by serving as a barrier and trapping site for pollutants^10^^,^^11^.

There are challenges in green buildings, such as creating an effective green environment and providing relaxing surroundings^12^.Air phytoremediation technology has many potential advantages to reduce pollutants in air and improve air quality^13^^,^they fix carbon dioxide through photosynthesis and help to decrease greenhouse gases in the atmosphere^14^, there is a need for removing pollution in high concentration by plants, particularly indoor air VOCs pollutants and Particulate matter was not studied in detail especially in pharmaceutical laboratory indoor air and. Reducing indoor pollution in pharmaceutical laboratories can be controlled by combination of biofiltration and phytoremediation results in the bio-wall technique^1^. Further research is needed before this technology becomes widely adopted and implemented within indoor environments. To further validate the potential of these systems for air quality remediation, reproducible laboratory and Feld experimentation is required to quantify the effects and variances with respect to system designs^15^.

**Fig. 1:**
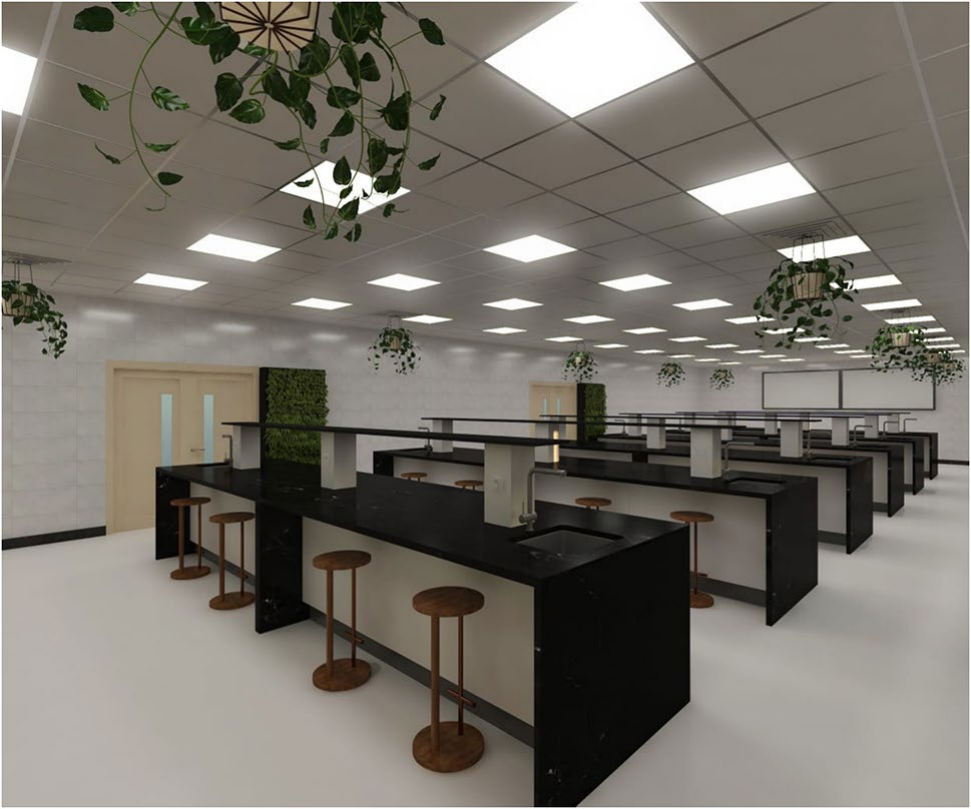
Green-integrated pharmaceutical laboratory interior with air-purifying plants.

This study investigates the combined effect of these factors by evaluating four ornamental plant species as phytoremediation agents using both pot-based and green wall systems^16^. By correlating their morphological and physiological features with VOC removal efficiency, this research provides an integrated model for selecting optimal indoor plant species for sustainable IAQ management in pharmaceutical environments. (Fig. 1)

## Materials and methods

### Plant materials (Experimental procedures)

#### Plant physiology

**Fig. 2:**
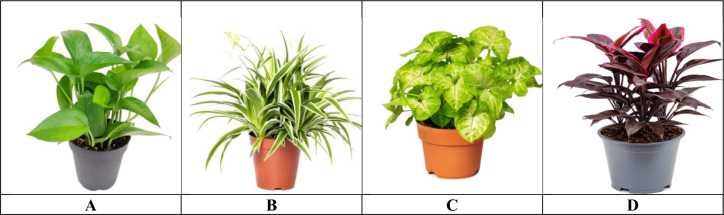
Ornamental indoor studied plants **A:** *Epipremnum aureum*, **B:** *Chlorophytum comosum,* **C:** *Syngonium podophyllum*, **D:** *Cordyline fruticosa*

The selected plant species A: Jade Pothos *Epipremnum aureum*, B: Spider Plant *Chlorophytum comosum*, C: *Syngonium podophyllum*, and D: *Cordyline fruticosa* were chosen based on their documented potential for indoor air purification and adaptability to low-light indoor environments (Fig. 2). *Epipremnum aureum* and *Syngonium podophyllum* belong to the Araceae family, characterized by broad leaves with high stomatal activity, which enhances gaseous pollutant uptake. *Chlorophytum comosum* and *Cordyline fruticosa*, belonging to the Asparagaceae family, are known for their tolerance to environmental stress and efficient removal of indoor pollutants such as benzene and formaldehyde. Morphologically, these species exhibit variations in leaf area, surface roughness, and wax content, which influence their ability to absorb particulate matter and absorb volatile organic compounds (VOCs). Scientifically, these plants have been widely reported for their phytoremediation capacity, including VOC absorption, CO₂ reduction, and particulate matter capture, making them suitable candidates for evaluating sustainable indoor air quality improvement strategies. A total of twelve pots for each species were sourced from plant suppliers (AASTMT plant nursery). The pots were filled with loamy soil consisting of approximately 30% sand, 30% silt, 15% clay, and 25% humus. The plants were acclimated to laboratory conditions for one month and watered as needed^17^. Before starting the experiments, the total leaf area and plant height for all species were measured.

#### Plant morphology

The leaves of all plants were classified into three categories: large, medium, and small, and then counted. Three sample leaves from each category were selected and their surface areas were measured using the ImageJ software (a Java-based processing program developed by the National Institutes of Health and the Laboratory for Optical and Computational Instrumentation)^18^. The average surface area of the sample leaves was calculated and then multiplied by the total number of leaves in each category. Finally, the total surface area was determined by summing the individual areas of all the leaves (Table 1). Plant height was measured before and at the end of the experiments. For fresh wet weight, three leaves weight were determined by digital electronic balance (4 Digital balance LAB254i – ADAM.UK) and reported in mg cm^2^ of leaf area, and dry weight of leaves were measured by an electronic balance, drying them in the oven (Binder ED-S 056 – Germany) under 80° C for 24 h, then weighing out the dried leaves, calculating and reporting in mg cm^2^ of leaf area.

**Table 1:** Plant species leaves number and total leaf area.

	***Epipremnum aureum***	***Chlorophytum comosum***	***Syngonium podophyllum***	***Cordyline fruticosa***
Small leaf no.	11	0	10	3
small leaf area cm^2^	40.331± 86.01	0	88.773± 85.163	45.571± 86.913
Medium leaf no.	5	12	3	7
medium leaf area cm^2^	50.989 ± 85.041	32.47± 85.163	93.574± 88.067	56.993 + 85.028
Large leaf no.	1	30	3	5
large leaf area cm^2^	73.261 ± 85.842	36.071± 89.611	95.038 ± 90.347	62.779± 87.098
Total leaf area cm^2^	771.392 cm^2^	1471.77 cm^2^	1453.566 cm^2^	849.559 cm^2^

#### Determination of Air pollution tolerance index (APTI)

To determine the Air Pollution Tolerance Index (APTI) for the four examined indoor plant species, four biochemical parameters were measured simultaneously during the experiments for each plant: total chlorophyll content, relative water content (RWC), leaf extract pH, and ascorbic acid content^19,20^. Each experiment was performed with three independent technical replicates (n = 3) under identical conditions, and the results are presented as mean ± SEM. All plant species were evaluated under controlled and consistent experimental conditions, with measurements conducted following a standardized protocol to ensure reproducibility. Furthermore, experimental runs were carefully scheduled to minimize variability associated with laboratory activity, chemical usage, occupancy, and ventilation conditions. Plant tolerance measures based on Air Pollution Tolerance Index (APTI) values (Table 2). Summarized as following categories^20^

**Table 2:** APTI Value Category^20^**.**

**APTI value**	**Response**
1–11	Sensitive
12–16	Intermediate
≥ 17	Tolerant

To calculate the APTI using the Equation (1)

1 $$\mathbf{A}\mathbf{P}\mathbf{T}\mathbf{I} =\frac{{\boldsymbol{A}}\left({\boldsymbol{T}}+{\boldsymbol{P}}\right)+\left({\boldsymbol{R}}\right)}{10}$$

Where, A= Ascorbic acid content (mg. g^-1^) (dry weight), T = Total chlorophyll content (mg. g^-1^),P = leaf extract pH, and R= RWC (%).

##### Total chlorophyll

A crushed leaf sample (5 g) was taken, and 10 mL of 80% acetone was added to it. Then, the sample was left aside for 15 min for thorough extraction. The leaf extract obtained was decanted into centrifuge tubes and was centrifuged using (Centrifuge – HETTCH-EBA 200) at 2500 rpm for 10 min. Then, the absorbance was taken at 645 nm and 663 nm using a spectrophotometer (Jenway-7300 UK) after calibrating it against reagent blank. The total chlorophyll content was calculated using equation (2)

2 $$\mathbf{T}\mathbf{C}\mathbf{h}=[20.2(\mathbf{A}645)+8.02(\mathbf{A}663)]\times [\frac{{\boldsymbol{V}}}{(1000\times {\boldsymbol{W}})}]$$

Where, TCh= total chlorophyll (mg. g^-1^), A645 = absorbance at 645 nm, A663 = absorbance at 663 nm, V = total volume of the extract (ml), and W = weight of the sample (g).

###### Relative water content (RWC)

Three distinct leaf weights, the fresh weight obtained right away after gathering the leaves (FW) and the turgid weight obtained after submerging the leaves in water for an entire night (TW) were measured for every plant under examination then measure the dry weight after drying in hot air oven at 70 °C overnight; (Hot air oven BINDER- ED-S 056- Germany) using 4-digit electronic balance (Aczet CY4102C). The measurements were done for triplicate and take the average value. For calculating the RWC of leaves equation (3) was used.

3 $$\mathbf{R}\mathbf{W}\mathbf{C}=\frac{{\boldsymbol{F}}{\boldsymbol{W}}-{\boldsymbol{D}}{\boldsymbol{W}}}{{\boldsymbol{T}}{\boldsymbol{W}}-{\boldsymbol{D}}{\boldsymbol{W}}} \hspace{0.17em}\times \hspace{0.17em}100\mathrm{\%}$$

Where, FW = fresh weight, DsW= dry weight, and TW = turgid weight.

###### Leaf-extract pH

For determining the pH for each examined plant, a part of the leaf sample (5 g) was crushed and homogenized using homogenizer (Homogenizer 300-25000 rpm - unidrive CAT1000) in 50 mL deionized distilled water. The sample was filtered, and the pH of leaf suspension was measured using a pH meter (Jenway 3510 pH-UK) average value is taken for three measurements.

###### Ascorbic acid

1 g of fresh leaves were sliced up and placed inside a test tube. Then, 4 mL oxalic-ethylenediaminetetraacetic acid extracting solution was added to the test-tube followed by 1 mL orthophosphoric acid, 1 mL of sulfuric acid (5%), 2 mL ammonium molybdate (5%) and 3 mL distilled water. The solution was allowed to stand for 15 min and the absorbance was measured at 760 nm with a spectrophotometer (Jenway-7300 UK). Using the ascorbic acid standard curve in mg mL^−1^, the ascorbic acid concentration was determined and then converted to ascorbic acid content per gram dry weight. Then determine the Ascorbic acid content for each examined plant and repeat three times and take the average value^19^.

#### Dust-capturing potential

To estimate the dust-capturing potential of the leaves: For each of the selected plant species, fresh mature leaves were collected during the morning hours (08:00) and measured the weight (W_1_ weight of leaves and dust). The sampling was done in triplicate. Then the leaves of the selected plant species were washed in the early morning (08:30) where the upper and lower surfaces of leaves were cleaned using a fine brush and distilled water. After the leaves were air-dried, a four-digit electronic balance (Aczet CY4102C), was used for the measurement of leaf weights (W_2_ weight of leaves without dust). The leaf area was measured using Image J software^18^.

The dust-capturing potential of leaves was calculated using equation (4)

4 $$\mathbf{W}=\frac{({\boldsymbol{W}}1-{\boldsymbol{W}}2)}{{\boldsymbol{A}}}$$

Where W= dust capturing potential (g.cm^-2^), W_1_= weight of leaf with dust (g), W_2_= weight of leaf after removing dust (g) and A= total area of leaf (cm^2^)^20^

#### Cuticle wax extraction

The amount of cuticle wax of each of the four potted plants was determined before exposure to the four VOCs compound and after the exposure. The method of cuticle wax extraction was adopted from^21^^,^^22^ method .The total surface area of leaves for each type of plant was around 130 cm^2^.The leaves of each type of plant were cut into small pieces (1 × 1 cm^2^) and put into a stoppered conical flask. Methanol and chloroform were used as the solvents for extraction at ratio of 1:1 by volume. Methanol (30 mL) and chloroform (30 mL) were poured into stoppered conical flask of each sample for complete extract of the wax, the prepared samples were shaken at 240 rpm for 8 h. using shaking incubator (IKA KS30001 Germany). After the shaking process, the solvents were put into a petri dish for evaporation for about 5-7 h in fume hood (Labtech fuming hood). The remaining part was only wax then determined the cuticle wax weight using sensitive digital balance (4 Digital balance LAB254i – ADAM.UK). Repeat three times and take the average weight.

#### Stomatal density and stomata count

The four examined plants were examined under microscope for stomata counting using Replica technique. For stomatal slide determination using the replica method where nail varnish was used to copy the pattern of stomata on the leaves, it was carried out in early time in the morning to optimize the opening of stomata The number of stomata per field of view (FOV) was converted to the number of stomata per one mm^2^ of leaf using a standard scale. The diameter of the FOV at 400x is around 4.5µm (0.45 mm). Counted the stomata in at least 5 FOVs on the lower leaf surface determined the average no. of stomata. A light microscope (Trinocular light microscope- B-193, Optica Italy) with 400× (ocular x objective) was applied to observe the appearance and count the number of stomata^10,11^. That stomatal number categorized into five groups: few (1-50), quite a few (51-100), many (101-201), many more (> 201), and infinite (> 300). Where Stomatal density was calculated using the formula:

Stomatal density (SD) = # of stomata /area of field of view (FOV)

Stomatal density can be grouped into three categories. Low density (<300/cm^2^), medium density (300-500/cm^2^) and high density (>500/cm^2^)^19^.

### Pharmaceutical organic laboratory experimental settings and measurements

To assess and validate the air-purification capabilities of selected plants, controlled experiments were conducted in a pharmaceutical organic chemistry laboratory with dimensions of 8.5 m (width) × 20.34 m (length) × 3.04 m (height), resulting in a total volume of approximately 525.59 m^3^. The study was carried out under real-world environmental conditions, both in the presence and absence of indoor plants. Air quality measurements were conducted during scheduled student practical sessions focusing on the identification of aromatic compounds, aldehydes, and ketones. VOC measurements were conducted under real operating conditions during practical sessions by Henan OC-1000 Multi-Gas Detector. The monitoring focused specifically on benzene and toluene, as these were the primary detectable compounds within the instrument’s selectivity range, while other solvents present in the laboratory were not detected by the device. These sessions were held under standard ventilation and exhaust system operations.

Twelve potted plants (two per laboratory bench) were strategically placed across six benches to ensure uniform distribution. Indoor air quality (IAQ) parameters including volatile organic compounds (VOCs), carbon monoxide (CO), carbon dioxide (CO₂), particulate matter (PM₂.₅ and PM₁₀) were monitored using the Henan OC-1000 Multi-Gas Detector, as described by Taheri and Hamzehlouyan^23^. Each experimental run lasted 40 minutes, corresponding to the duration of a typical laboratory session. To ensure accuracy and repeatability, the removal efficiency measurements were conducted in triplicate under identical conditions.

#### Removal efficiency and IAQ measurements

The efficiency of the plants in removing these contaminants was determined by calculating the percentage reduction in concentration. The initial concentration (C_0_) of the contaminants in the lab (without plants) was compared to the final concentration (C_n_) after introducing the plants for the same experiment duration under the same conditions (ventilation, number of students, chemicals used). Therefore, the removal efficiency (RE%) was calculated for each IAQ parameters using the equation (5)^24^

5 $${\mathbf{RE}}\% = \, \left( {{\mathbf{C}}_{{\mathbf{0}}} - \, {\mathbf{Cn}}} \right) \, / \, {\mathbf{C}}_{{\mathbf{0}}} \times \, {\mathbf{100}}$$

#### Multivariate statistical chemometric analysis for evaluating plant species efficiency

An exploratory analysis of the data composed the removal rate of different air pollutants i.e. TVOCs, CO_2_, CO and particulate matter PM_10_, PM_2.5_ by the plants under study (*Cordyline fruticose* (Co)*, Epipremnum aureum* (JP)*, Syngonium podophyllum* (Sy)*, Chlorophytum comosum* (SP), was carried out using principal component analysis (PCA) is a statistical technique used in data analysis to reduce the dimensionality of large datasets, while retaining most of the variation present in the data ,and hierarchical clustering analysis (HCA) is a method of cluster analysis that groups similar data points or objects into clusters or “hierarchies” based on their similarity. Unlike other clustering techniques, hierarchical clustering builds a tree-like structure called a dendrogram, where each node represents a cluster of points. OPLS-DA model presented in this study was applied as an exploratory multivariate tool to visualize patterns among plant species rather than to develop a definitive predictive classification model. Given the relatively limited sample size, the possibility of model overfitting cannot be excluded. The observed clustering trends primarily reflect underlying variations in physiological and phytoremediation-related traits rather than absolute group separation. Accordingly, the OPLS-DA outputs are used to support data interpretation and highlight potential relationships among variables, rather than to draw strong predictive conclusions. This approach ensures a more conservative and statistically responsible interpretation of the multivariate analysis.

The analysis aimed to display the variabilities in the efficiency of the four plants in phytoremediation of the studied air pollutants. On the other hand, supervised pattern recognition method, orthogonal projection to latent structures-discriminant analysis (OPLS-DA), was performed to highlight the possible correlation existing between the X variables (the different plants) and Y variables (the morphological and physiological indices of the plants). Permutation analysis was carried out to further confirm the significance and validity of the OPLS-DA model, respectively. Data were analyzed by using SIMCA-P software (Version 14.0, Umetrics, and Umeå, Sweden).

### Data analysis

Data analysis was performed using the IBM SPSS software package version 20.0 (Armonk, NY: IBM Corp). Quantitative data were presented as a range (minimum and maximum), along with mean, standard deviation, standard error, median, and interquartile range (IQR). The results were considered statistically significant at a 5% confidence level. The Kruskal-Wallis H test indicated significant differences among the plant groups (p < 0.001), demonstrating that incorporating indoor plants impacts the reduction of air pollutants. For normally distributed quantitative variables, an F-test (ANOVA) was used to compare more than two groups, and pairwise comparisons were made using the Post Hoc Tukey test.

## Results and discussions

### Plant morphology and physiology measurements

As demonstrated in (Table 2), Plant height ranged from 17 cm for *Epipremnum aureum* to 59 cm for *Cordyline fruticosa*. Fresh wet weight of three leaves varied across the species and Dry weight of three leaves was highest for *Cordyline fruticosa* and *Syngonium podophyllum*.

#### Air pollution tolerance index (APTI)

**Fig. 3:**
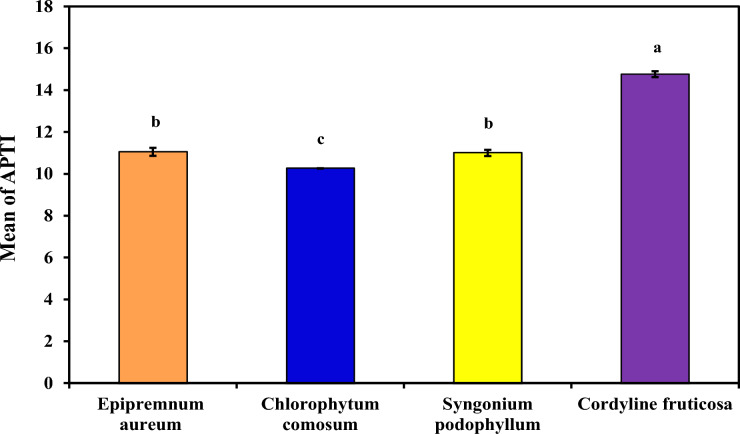
Comparison between examined plants according to their APTI.

As demonstrated in (Table 3 and Fig. 3) which demonstrated Air pollution tolerance index (APTI) in each plant extract examined (*Epipremnum aureum*, *Chlorophytum comosum*, *Syngonium podophyllum* and *Cordyline fruticosa*). The ranking of plant species tolerant to indoor air pollution with the APTI values among the four plant species according to the experimental analysis is as follows: *Cordyline fruticose* (14.76%14.76^a^ ± 0.14)** >** *Epipremnum aureum* (11.05%11.05^b^ ± 0.19) > *Syngonium podophyllum* (11.0 %11.0^b^ ± 0.15) > and *Chlorophytum comosum* (10.26%10.26^c^ ± 0.006). *Cordyline fruticosa* demonstrated the highest APTI (14.76%). Higher APTI values suggest greater suitability of a species for use in polluted environments; they should not be interpreted as a direct proxy for VOC or particulate matter removal capacity. In this context, APTI serves as a screening tool to identify tolerant species that may be further evaluated for their phytoremediation potential using direct pollutant uptake measurements. Recent studies further support the importance of integrating physiological resilience with functional phytoremediation performance when evaluating plant-based air purification systems. Emerging research highlights that plants exposed to environmental pollutants not only act as passive filters but also activate complex biochemical pathways, including the production of plant-derived bioactive compounds with antioxidative and detoxifying roles^25^. These compounds contribute to mitigating oxidative stress induced by prolonged exposure to hazardous pollutants such as VOCs. In parallel, long-term exposure studies emphasize that continuous contact with environmental contaminants can induce adaptive physiological responses, including alterations in chlorophyll content, enzymatic activity, and stress-related metabolites, which directly influence plant health and pollutant removal capacity^26^. Furthermore, recent findings on plant–pollutant interactions demonstrate that structural and biochemical traits such as cuticular wax composition, stomatal behavior, and metabolic transformation pathways play a crucial role in pollutant uptake, sequestration, and degradation processes^27^. These insights align with the current study, where variations in physiological traits (e.g., APTI components, stomatal density, and wax content) were closely associated with differences in phytoremediation efficiency among species. Collectively, these studies reinforce the need for a multidimensional evaluation framework that combines physiological tolerance, biochemical response, and direct pollutant removal metrics to more accurately assess and optimize plant-based biofiltration systems for indoor air quality improvement.

**Table 3:** Morphological parameter for examined plants.

	***Epipremnum aureum***	***Chlorophytum comosum***	***Syngonium podophyllum***	***Cordyline fruticosa***
Plant height	*17cm*	*26 cm*	*55 cm*	*59 cm*
Fresh wet weight of three leaves	*4.6392 gm*	*7.3361 gm*	*4.7537 gm*	*3.6242 gm*
Dry weight of three leaves	*0.3455 gm*	*0.6610 gm*	*0.6838 gm*	*0.8025 gm*

#### Total chlorophyll content

**Fig. 4:**
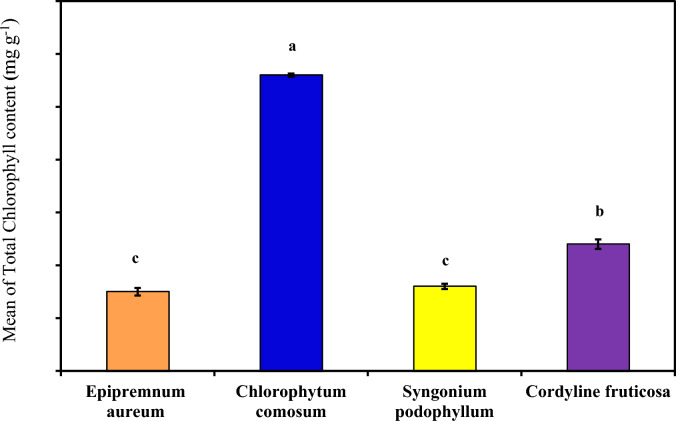
Comparison between the different studied groups according to total chlorophyll.

As demonstrated in (Table 4 and Fig. 4) which demonstrated Total chlorophyll content in each plant extract examined (*Epipremnum aureum*, *Chlorophytum comosum*, *Syngonium podophyllum* and *Cordyline fruticosa*). *Chlorophytum comosum* demonstrated the highest Total chlorophyll content 0.056^a^ ± 0.0003 followed by *Cordyline fruticosa*, *Syngonium podophyllum* and *Epipremnum aureum*, with mean equal to (0.056^a^ ± 0.0003, 0.024^b^ ± 0.0009, 0.016^c^ ± 0.0005 and 0.015^c^ ± 0.0007 mg g^-1^ respectively). In the current investigation, the total chlorophyll contents of all examined plant species were comparatively lower. This can be a result of exposure to air pollutant. As TCH An indicator of photosynthetic activity, growth, and biomass productivity; a decrease in chlorophyll content indicates that plants are more sensitive to air pollution, and degradation of photosynthetic pigments has long been used as an indication of air pollution. Same observation confirmed my^20^^,^^28^, Therefore, *Epipremnum aureum*, *Syngonium podophyllum* were likely to be more sensitive to air pollution than *Chlorophytum comosum* and *Cordyline fruticosa*.

**Table 4:** Comparison between examined plants according to their total Chlorophyll, APTI and Dust Capturing.

	Total chlorophyll content (mg g^-1^)	APTI (%)	Dust capture potential (× 104) (gcm^−2^)
*Epipremnum aureum*	*0.015c* ± *0.0007(0.0004)*	*11.05b* ± *0.19 (0.11)*	*1.35b* ± *0.21 (0.12)*
*Chlorophytum comosum*	*0.056a* ± *0.0003(0.0002)*	*10.26c* ± *0.006 (0.003)*	*9.52a* ± *4.2 (2.4)*
*Syngonium podophyllum*	*0.016c* ± *0.0005(0.003)*	*11.0b* ± *0.15 (0.08)*	*1.63b* ± *0.30 (0.17)*
*Cordyline fruticosa*	*0.024b* ± *0.0009(0.0005)*	*14.76a* ± *0.14 (0.08)*	*4.04ab* ± *3.83 (2.21)*
F	2456.226*	658.721*	5.316*
p	< 0.001*	< 0.001*	0.026*

3 Replica for each group Data was expressed using Mean ± SD. (SE)

SD: Standard deviationSE: Standard error

F: F for One way ANOVA test, Pairwise comparison bet. Each 2 groups was done using Post Hoc Test (Tukey)

p: p value for comparing between the three different studied time *: Statistically significant at p ≤ 0.05

##### Relative water content (RWC)

**Fig. 5:**
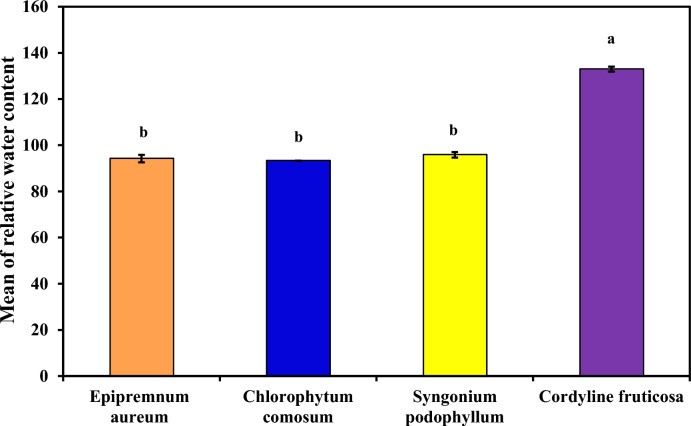
Comparison between examined plants according to RWC.

As demonstrated in (Table 5 and Figure 5) which demonstrated Relative water content (RWC) in each plant extract examined (*Epipremnum aureum*, *Chlorophytum comosum*, *Syngonium podophyllum* and *Cordyline fruticosa*). *Cordyline fruticosa* demonstrated the highest Relative water content followed by *Syngonium podophyllum*, *Epipremnum aureum* and *Chlorophytum comosum*, with mean equal to 133.0^a^ ± 1.10 %, 95.86^b^ ± 1.22 %, 94.21^b^ ± 1.65 % and 93.30^b^ ± 0.04% respectively. The four plant species having high RWC%. Under stressful circumstances, as exposure to indoor air pollution or dryness days, these plants may transpire and survive more frequently. in *Cordyline fruticosa*, which is a succulent ornamental species with high water storage capacity, the leaf tissue can retain substantial free water under ambient laboratory conditions. In addition, slight underestimation of full turgid weight (TW), which is commonly influenced by equilibration time and osmotic adjustment during rehydration, may result in FW values marginally exceeding TW, leading to RWC values slightly above 100%. Similar observations have been reported in previous physiological studies for thick-leaved or succulent plant species, where RWC values marginally exceed 100% due to methodological constraints in defining absolute full turgidity. The results for RWC in the present study are inconformity with the results of^20^^,^^28^^,^^29^.

**Table 5:** Comparison between examined plants according to Ascorbic acid content, pH and RWC.

	**Ascorbic acid (mg g**^**-1**^**)**	**PH leaf extract**	**Relative water content (%)**
*Epipremnum aureum*	*1.69a ± 0.02 (0.01)*	*9.67a ± 0.18 (0.10)*	*94.21b ± 1.65 (0.95)*
*Chlorophytum comosum*	*0.98d ± 0.01 (0.004)*	*9.40ab ± 0.05 (0.03)*	*93.30b ± 0.04 (0.02)*
*Syngonium podophyllum*	*1.51c ± 0.03 (0.018)*	*9.39ab ± 0.49 (0.28)*	*95.86b ± 1.22 (0.70)*
*Cordyline fruticosa*	*1.61b ± 0.02 (0.009)*	*8.70b ± 0.18 (0.11)*	*133.0a ± 1.10 (0.64)*
F	682.475*	6.683*	845.089*
p	<0.001*	<0.014*	<0.001*

3 Replica for each group Data was expressed using Mean ± SD. (SE)

SD: Standard deviationSE: Standard error

F: F for One way ANOVA test, Pairwise comparison bet. each 2 groups was done using Post Hoc Test (Tukey)

p: p value for comparing between the three different studied time *: Statistically significant at p ≤ 0.05

###### Leaf-extract pH

**Fig. 6:**
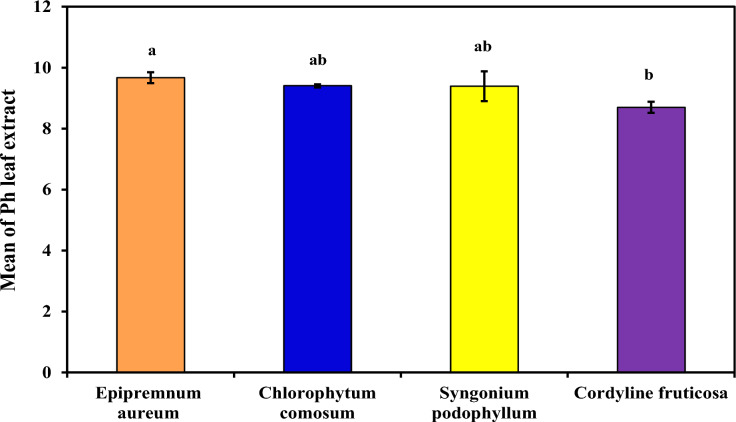
Comparison between examined plants according to pH.

As demonstrated in (Table 5 and Figure 6), which demonstrated Leaf-extract pH in each examined plant extract (*Epipremnum aureum*, *Chlorophytum comosum*, *Syngonium podophyllum* and *Cordyline fruticosa*). The four plant species demonstrated high Leaf-extract pH (more alkaline), with mean equal to (9.67^a^ ± 0.18, 9.40^ab^ ± 0.05, 9.39^ab^ ± 0.49 and 8.70^b^ ± 0.18 respectively). The findings demonstrated that certain plant species from the contaminated area had an acidic pH, and others had a basic pH, *Cordyline fruticosa* has a lower leaf-extract pH at 8.70, suggesting better tolerance to acidic pollutants. *Epipremnum aureum*, *Syngonium podophyllum*, and *Chlorophytum comosum* have higher leaf-extract pH values, indicating a potential sensitivity to acidic pollutants. When an acidic pollutant is present, the pH of the leaf’s decreases, and in sensitive plants, this reduction occurs more quickly than in tolerant plants. Similar finding by (Pandit, 2017). In line with (Hazarika, 2023) Plants establish their detoxifying system at an alkaline ph. Hence, trees are regarded as tolerant species when leaf extracts reach a neutral or alkaline pH as a result, plants that have high leaf extract pH are more resilient to air pollution.

###### Ascorbic acid

**Fig. 7:**
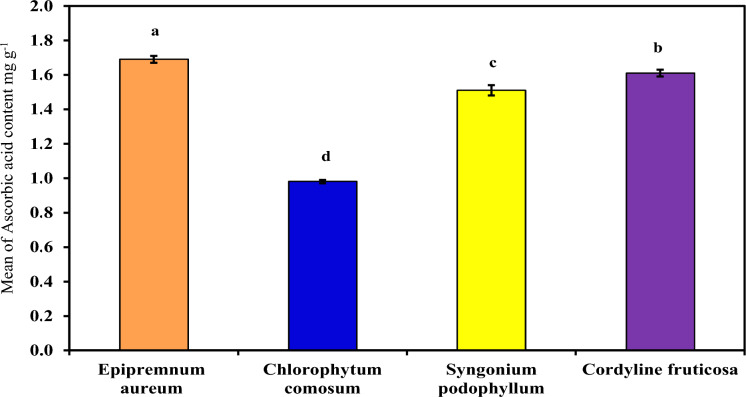
Comparison between examined plants according to their Ascorbic acid content.

As demonstrated in (Table 5 and Figure 7) which demonstrated Ascorbic acid concentration in each examined plant extract (*Epipremnum aureum*, *Chlorophytum comosum*, *Syngonium podophyllum* and *Cordyline fruticosa*). Both *Cordyline fruticosa* and *Epipremnum aureum* demonstrated the highest ascorbic concentration followed by, *Syngonium podophyllum* and *Chlorophytum comosum*, with mean equal to 1.69^a^ ± 0.02, 1.61^b^ ± 0.02, 1.51^c^ ± 0.03 and 0.98^d^ ± 0.01 mg g^-1^ respectively). Ascorbic acid’s content likewise varies with pH, being higher at higher pH values and lower at lower pH values. *Cordyline fruticosa* and *Epipremnum aureum* had the highest ascorbic acid concentration, indicating their ability to combat oxidative stress caused by air pollution. *Syngonium* and *Chlorophytum comosum* had lower concentrations. The results for ascorbic acid content are well tested by many other findings^28^^,^^20^^,^^29^.

Given its high APTI, total chlorophyll content, RWC, ascorbic acid content, and modest dust capture potential, *Cordyline fruticosa* appears to be a plant with potential for enhancing indoor air quality and mitigating indoor air pollution. Even so*, Chlorophytum comosum* also performs admirably when it comes to the crucial component of air purification dust capture potential.

In this study, both physiological tolerance indicators (APTI, RWC, chlorophyll content) and direct pollutant removal measurements were integrated to provide a comprehensive evaluation of phytoremediation performance. This combined approach allows not only the identification of species capable of surviving under polluted conditions but also those that actively contribute to air purification. The integration of these two perspectives demonstrates that *Cordyline fruticosa* exhibits both high physiological resilience and strong pollutant removal capacity, supporting its superior overall performance. This dual evidence strengthens the conclusion that *Cordyline fruticosa* is not only tolerant to indoor air pollution stress but also highly efficient in improving indoor air quality, making it a promising candidate for phytoremediation applications.

#### Dust-capturing potential

**Fig. 8:**
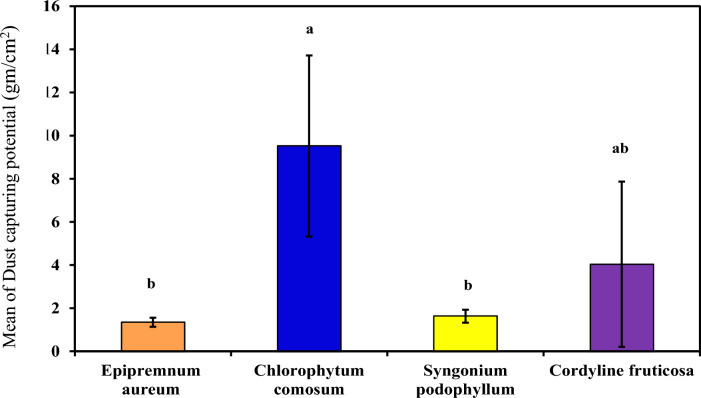
Comparison between examined plants according to their Dust capturing potential.

As demonstrated in (Table 4 and Fig. 8) which demonstrated Dust-capturing potential in each examined plant extract (*Epipremnum aureum*, *Chlorophytum comosum*, *Syngonium podophyllum* and *Cordyline fruticosa*). ***Chlorophytum comosum*** demonstrated the highest Dust-capturing potential 9.52^a^ ± 4.2×10^4^ gcm^−2^ indicating efficient dust trapping capabilities. *Cordyline fruticosa* and *Syngonium podophyllum* also show significant dust capture potential, while *Epipremnum aureum* has a lower value (4.04^ab^ ± 3.83, 1.63^b^ ± 0.30 and 1.35^b^ ± 0.21×10^4^ gcm^−2^ respectively). This finding correlates with H. Gawrońska et al. 2015^5^ results, that *Chlorophytum comosum* could reduce PM from indoor environments ranging from (13.62 - 19.79 μg/cm^2^). Another study conducted by S.^30^ that examined the effectiveness of *Chlorophytum comosum* with other species revealed its capacity (4.7–13 μg/cm^2^) for dust capturing potential^30^.

#### Cuticle wax extraction content

**Fig. 9:**
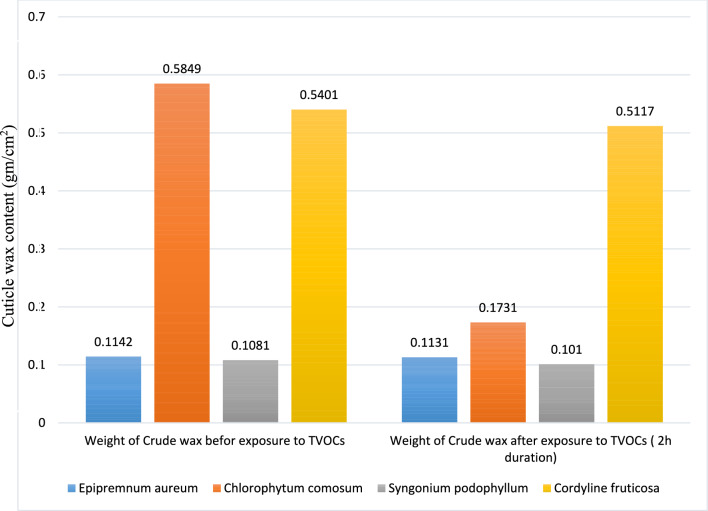
Cuticle wax content in examined plant extract.

The amount of cuticle wax of all four potted plants was determined before exposure for the VOCs and after the exposure. As demonstrated in (Table 6 and Fig. 9) demonstrated that Cuticle wax extraction weight for each examined plant (*Epipremnum aureum*, *Chlorophytum comosum*, *Syngonium podophyllum* and *Cordyline fruticosa*) before and after exposure to mixture of VOCs (0.5 ml from each benzene, toluene, benzaldehyde and Acetophenone). Our study demonstrated that there is significantly an increase in Cuticle wax extraction in *Chlorophytum comosum* (0.5849 gm/cm^2^) followed by *Cordyline fruticose* (0.5401 gm/cm^2^), *Epipremnum aureum* (0.1142 gm/cm^2^) and *Syngonium podophyllum* (0.1081 gm/cm^2^). However, after exposure to TVOCs for two hours, there was a notable decrease in cuticle wax content across all plant species, with *Chlorophytum comosum* showing the largest decrease (0.1731 gm/cm^2^), followed by *Cordyline fruticose* (0.5117 gm/cm^2^).

**Table 6:** Comparison between the weight of cuticle wax before and after exposure to TVOCs.

**Examined plant**	**Weight of Crude wax before exposure to TVOCs (gm/cm**^**2**^**)**	**Weight of Crude wax after exposure to TVOCs (2h duration) (gm/cm**^**2**^**)**
*Epipremnum aureum*	*0.1142*	*0.1131*
*Chlorophytum comosum*	*0.5849*	*0.1731*
*Syngonium podophyllum*	*0.1081*	*0.101*
*Cordyline fruticosa*	*0.5401*	*0.5117*

According to the data illustrated above, implying that TVOCs have a minimal impact on this plant’s action in manufacturing wax, *Epipremnum aureum* shows only small inhibition in wax formation after exposure. On the same note, though *Chlorophytum comosum* displays a huge reduction in wax production, this brings high sensitivity to VOCs. A study by^21^ revealed Chlorophytum comosum Cuticle Wax Content (0.81 mg/cm^2^), placing it in the moderate range compared to other plants in his study like *Dracaena sanderiana* with 7.06 mg/cm^2^). Partial effect to the chemicals was demonstrated by *Syngonium podophyllum* as it exhibited a moderate decrease in the wax contents. This fact correlates with earlier findings that the wax cuticle of the plant leaves can effectively absorb as well as concentrate benzene, one of the most typical SVOCs^31^^,^^32^. *Cordyline fruticosa* has a lower degree of sensitivity to TVOCs, which loses a small amount of wax after exposure to TVOCs. The findings prove the variability of plant species’ response to volatile organic compounds. The current study observed that, irrespective of the exposure to VOCs, *Cordyline fruticosa* had higher cuticle wax. This indicates that the plant is viable to remove VOCs for the long-distance extent in eliminating these pollutants. It still possesses a reasonable capacity for pollutant absorption due to its wax layer. This supports its effectiveness in indoor air quality improvement applications and sustainability in eliminating VOCs over an extended length of time. These findings represent novel study findings.

#### Stomatal density and stomata count

**Fig. 10:**
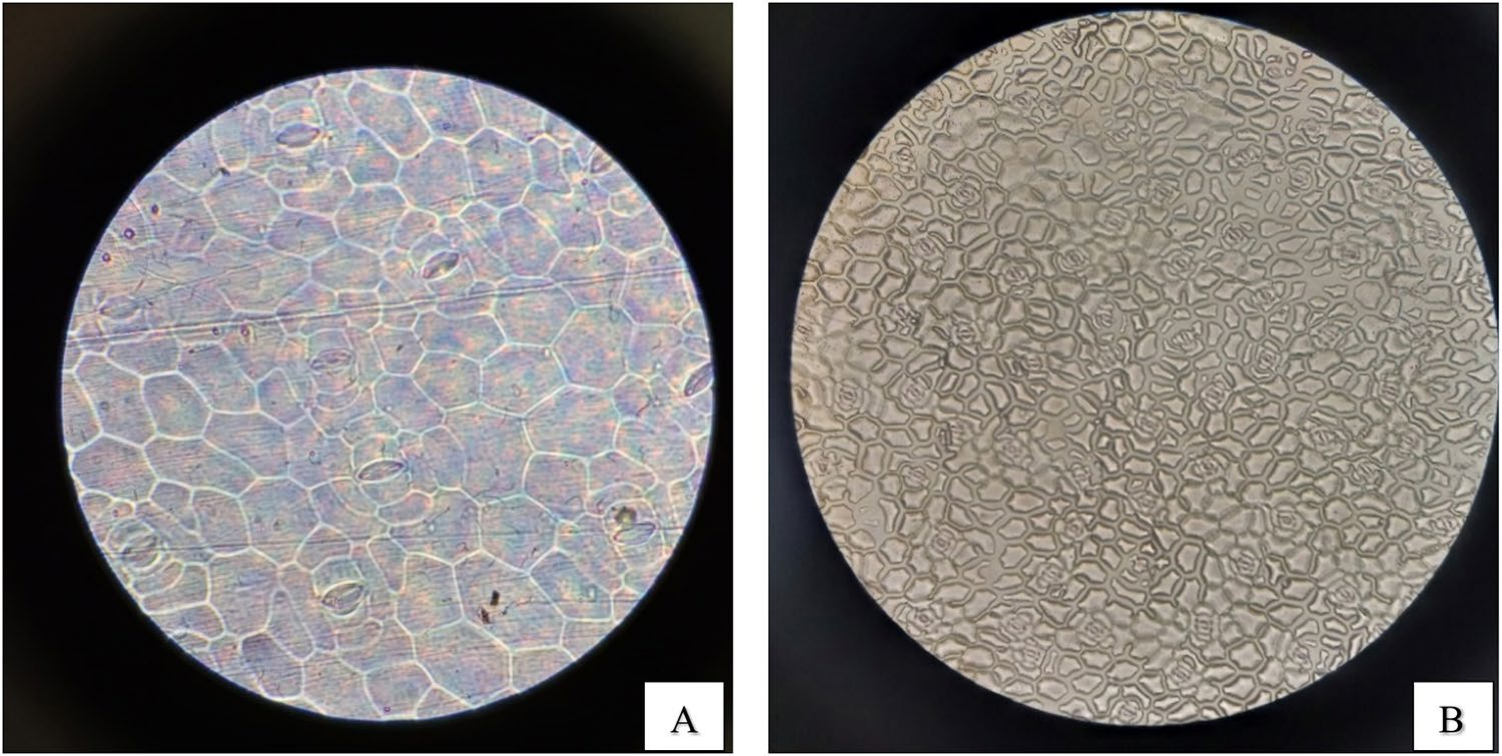
Observation of number of *Epipremnum aureum* stomata of lower surface using a microscope with (**A**) magnification 40×10 – (B) magnification 10×10.

**Fig. 11:**
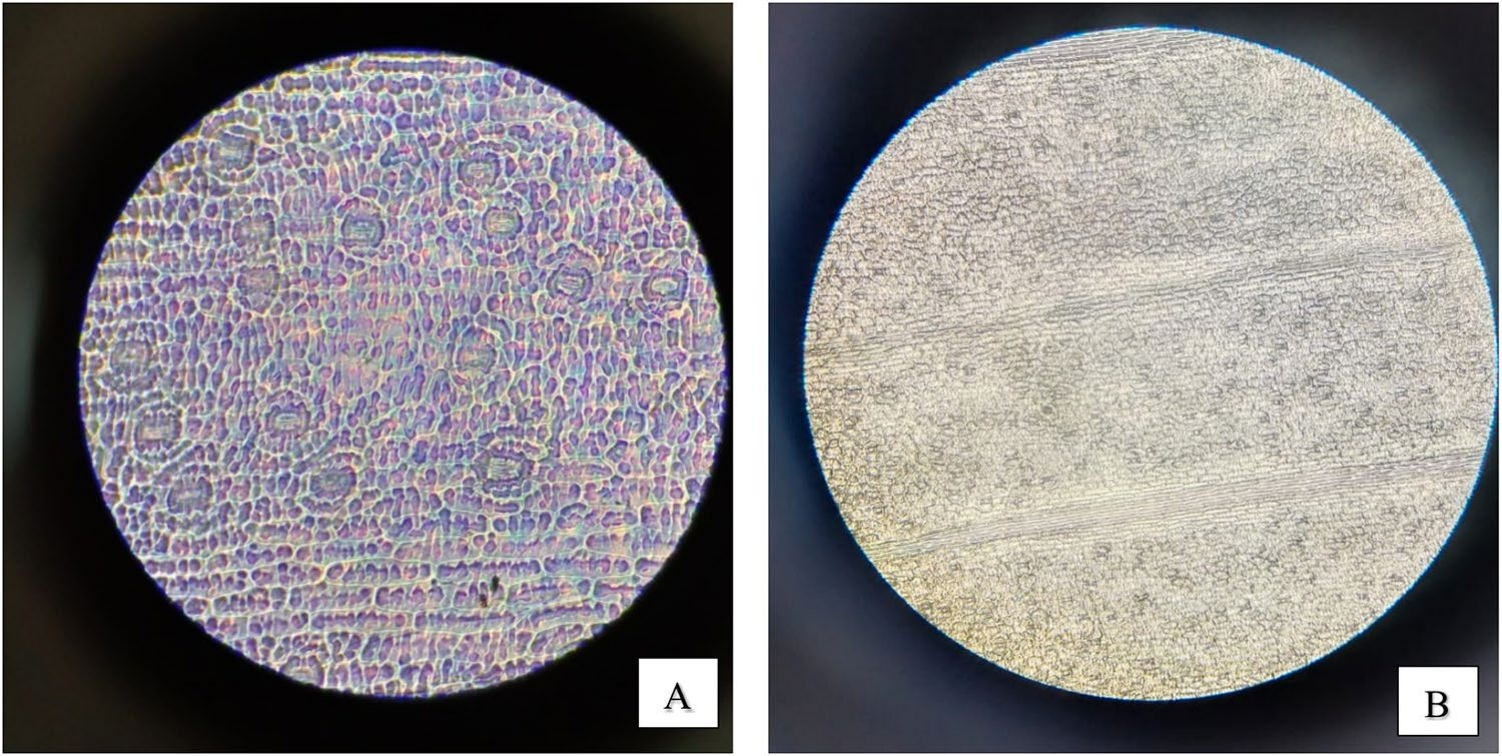
Observation of number of *Cordyline fruticosa* stomata of lower surface using a microscope with (**A**) magnification 40×10 – (**B**) magnification 10×10.

**Fig. 12:**
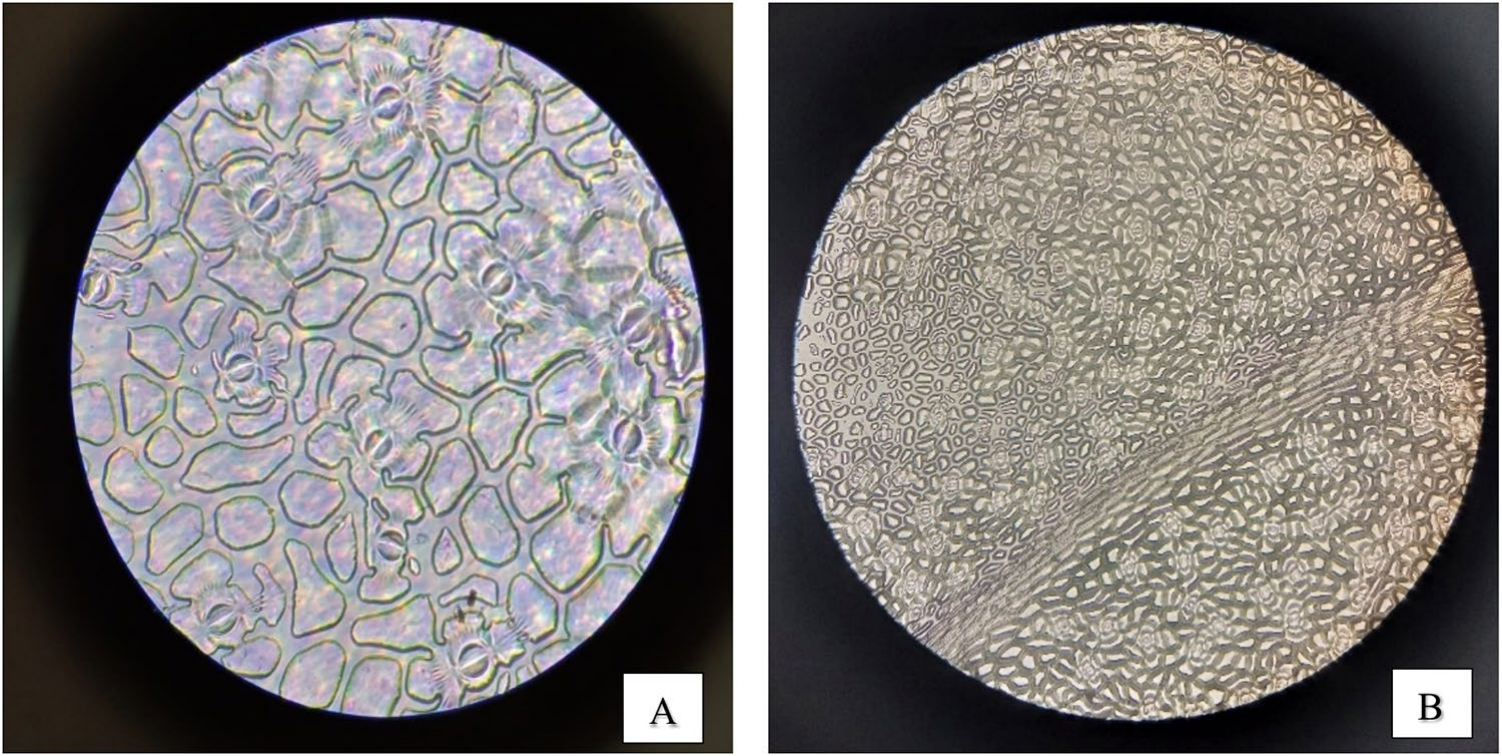
Observation of number of *Syngonium podophyllum* stomata of lower surface using a microscope with (**A**) magnification 40×10 – (**B**) magnification 10×10.

**Fig. 13:**
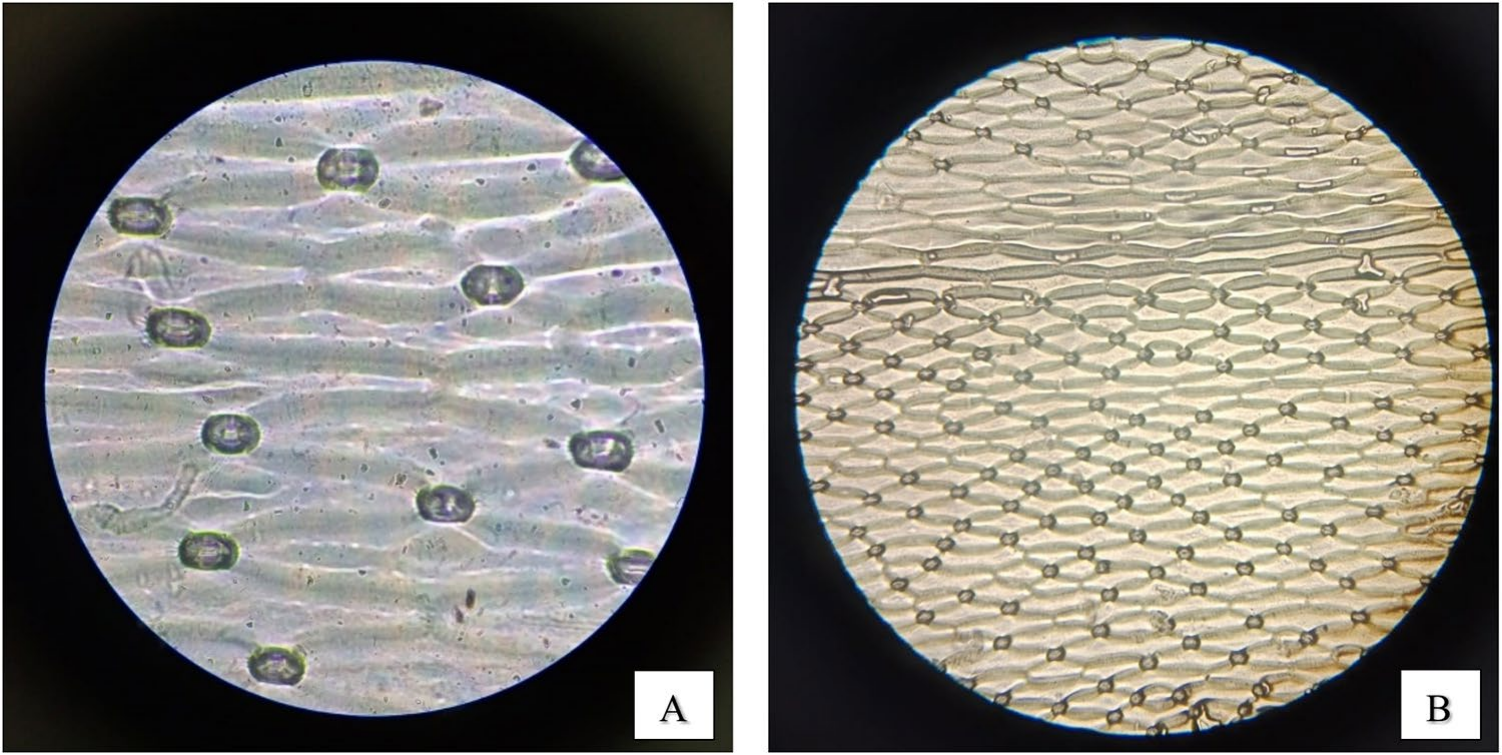
Observation of number of Chlorophytum comosum stomata of lower surface using a microscope with (**A**) magnification 40×10 – (**B**) magnification 10×10.

**Fig. 14:**
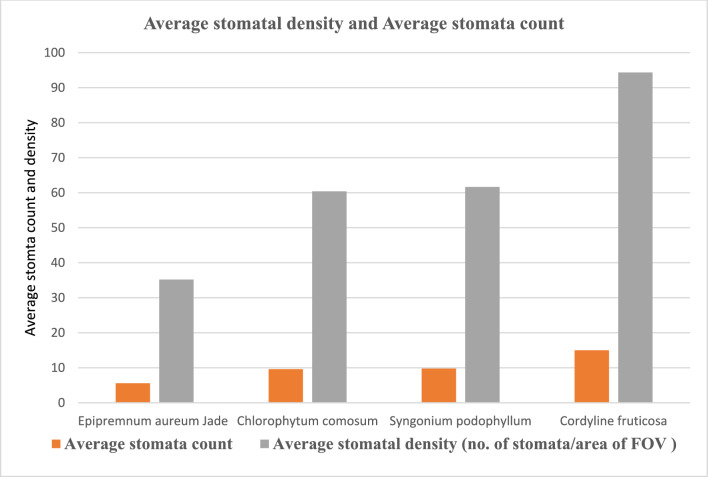
Examined plants stomata count and density distribution.

As demonstrated in (Figs. 10, 11, 12, 13) the evaluation of the stomatal density and stomatal count in the four species of plants under study, using the replica technique at 400 magnifications (corresponding to a 0.159 mm^2^ field of view area), indicates differences in these parameters. Table 7 and (Fig. 14) illustrated that *Epipremnum aureum* has a total leaf area of 50.98 cm^2^ and an average stomata count of 5.6 per field of view (FOV) area of 0.159 mm^2^. With an average stomatal density of 35.22 stomata/mm^2^, the density is modest. With a total leaf area of 36.47 cm^2^, *Chlorophytum comosum* has an average stomata count of 9.6 per FOV area of 0.159 mm^2^. The average stomatal density is 60.38 stomata/mm^2^, which indicates a moderate density. With a total leaf area of 93.57 cm^2^, *Syngonium podophyllum* has an average stomata count of 9.8 per FOV area of 0.159 mm^2^. Additionally demonstrating a moderate density, the average stomatal density is 61.63 stomata/mm^2^.

**Table 7:** Distribution of stomatal count and stomatal density of examined plants.

	***Epipremnum aureum***	***Chlorophytum comosum***	***Syngonium podophyllum***	***Cordyline fruticosa***
Average Stomata count in FOV	*5.6*	*9.6*	*9.8*	*15*
FOV area mm^2^	*0.159*	*0.159*	*0.159*	*0.159*
Total leaf area (cm^2^)	*50.98*	*36.47*	*93.57*	*56.99*
Average Stomatal density Stomata/mm^2^	*35.22*	*60.38*	*61.63*	*94.34*
Categories	*Low density*	*Moderate density*	*Moderate density*	*High density*
Number of stomata/ leaf area cm^2^	*1795.5*	*2201.9*	*5884.9*	*5089.6*
Categories	*Many*	*Many*	*Infinity*	*Infinity*

With a total leaf area of 56.99 cm^2^, *Cordyline fruticosa* has the largest average stomata count, with 15 per FOV area of 0.159 mm^2^. There are a lot of stomata; the average stomatal density is 94.34 stomata/mm^2^. In conclusion, among the plant species investigated, *Cordyline fruticosa* has the maximum stomata count and density, followed by *Syngonium podophyllum* and *Chlorophytum comosum*. In comparison to the other species, *Epipremnum aureum* has the lowest stomata count and density, indicating a reduced stomatal activity.

This is in concordance with other findings pointing to the fact that stomatal conductance greatly affects the uptake rate of VOCs by the leaf (Tani, Kato et al., 2007). Investigations made on the light-driven qualitative plant organs have explained the efficiency of formaldehyde, benzene, and toluene elimination from indoor air; essentially, stomata are involved in VOC absorption because they remain open in the presence of light^33^. In addition, regarding the leaf parameters, stomatal characteristics, cuticle wax layer components, hair growth affects the diffusion of VOCs in the leaves jointly. Variations in the ability to accumulate or sequester VOCs may also exist plant species may exist internally^34^,Kim et al., 2018).

### Real laboratory measurements for IAQ parameters (VOCs, CO, CO_2_, PM_2.5_ and PM_10_) presence and absence of the indoor plants

**Fig. 15:**
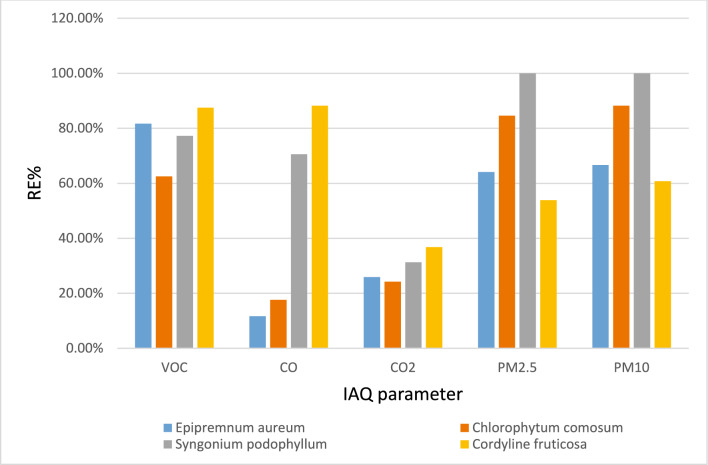
Removal efficiency RE% of examined plant for IAQ parameters in pharmaceutical organic chemistry Lab.

As demonstrated in (Table 8) and (Fig. 15) all four plant species contributed to the improvement of indoor air quality, but their efficiencies varied across different pollutants. *Cordyline fruticosa* exhibited the highest removal efficiency for volatile organic compounds (VOCs) at 87.5%, followed by *Syngonium podophyllum* (81.69%), *Chlorophytum comosum* (77.23%), and *Epipremnum aureum* (62.5%). In terms of carbon monoxide (CO) reduction, *Cordyline fruticosa* again showed the greatest efficiency (88.23%), while *Syngonium* followed closely (70.58%). Both *Chlorophytum* and *Epipremnum* were significantly less effective in CO removal, with efficiencies of 17.64% and 11.7%, respectively. For carbon dioxide (CO₂), *Cordyline* maintained its lead with a removal efficiency of 36.78%, with *Syngonium* (31.27%), *Epipremnum* (25.91%), and *Chlorophytum* (24.24%) trailing. The most striking results were observed in the reduction of particulate matter. *Syngonium podophyllum* achieved complete removal of both PM_2.5_ and PM_10_ up to 100% efficiency, highlighting its exceptional dust-capturing potential. *Chlorophytum comosum* also performed well in this category, removing 84.61% of PM_2.5_ and 88.23% of PM_10_. In contrast, *Epipremnum aureum* and *Cordyline fruticosa* displayed moderate particulate matter removal efficiencies.

**Table 8:** Removal efficiency RE% of examined plant for IAQ parameters in pharmaceutical organic chemistry Lab.

	**VOC** (ppm)	**CO** (ppm)	**CO**_**2**_ (ppm)	**PM**_**2.5**_ **(µg/m**^**3**^**)**	**PM**_**10**_ **(µg/m**^**3**^**)**
Final Conc. without plant C0	2.24	1.7	598	39	51
Final conc. with *Epipremnum aureum* Cf	0.84	1.5	443	14	17
*Epipremnum aureum* RE%	62.5%	11.7%	25.91%	64.10%	66.66%
Final conc. with *Chlorophytum comosum* Cf	0.51	1.4	453	6	6
*Chlorophytum comosum* RE%	77.23%	17.64%	24.24%	84.61%	88.23%
Final with *Syngonium podophyllum* Cf	0.41	0.5	411	0	0
*Syngonium podophyllum* RE%	81.69%	70.58%	31.27%	100%	100%
Final conc. with *Cordyline fruticosa* Cf	0.28	0.2	378	18	20
*Cordyline fruticosa* RE%	87.5%	88.23%	36.78%	53.84%	60.78%

These findings are consistent with previous studies that highlight the role of leaf structural characteristics, stomatal density, and cuticular wax in determining pollutant removal efficiency. Each species of plant has a special advantage when it comes to improving indoor air quality which is in line with experimental evidence showing that *Epipremnum aureum* had a greater benzene removal rate than *Chlorophytum comosum* (Song, 2011;^35,36^. While a small number of studies^37–39^ have demonstrated the effectiveness of potted plants in reducing the concentration of volatile organic compounds (VOCs) in virtually sized rooms, very few studies have examined the indoor quality of university pharmaceutical laboratories^40,41^. Prior research^10,42^^,^Irga,^43,44^,Kim, Khalekuzzaman et al. 2018,^45^ has assessed the potential of indoor potted plants to lower VOCs. Regarding to both research studies by Raghda Ramadan Abd- elaziz, T.M.El-keiy, E.A. Shalaby and A.M. Shehata^46^ and Su Y-M,^47^, they revealed that pothos only wall gardens succeeded in reducing CO_2_ by about 51%, and Syngonium only wall gardens succeeded in reducing CO_2_ by about 41%, while Pothos + Syngonium wall gardens succeeded in reducing CO_2_ by about 42 %^46^^,^^12^^,^^48^ this is supported by the conclusions drawn from current study investigation.

### Multivariate analysis of the plant phytoremediation efficiency

**Fig. 16:**
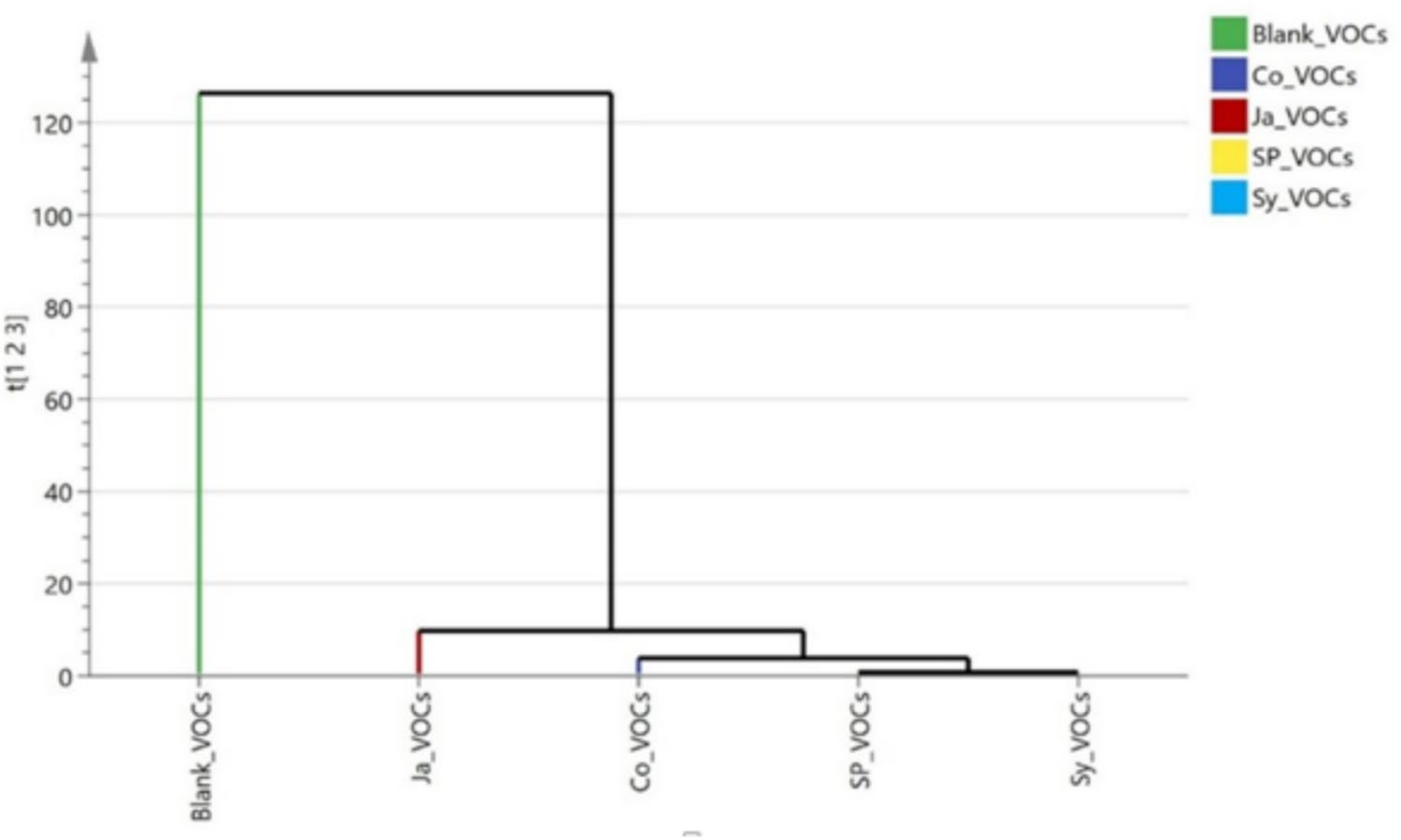
Hierarchical clustering (HCA) plot of the rate of removal of VOCs by the studied plants (*Cordyline fruticose* (Co)*, Epipremnum aureum* (Ja)*, Syngonium podophyllum* (Sy)*, Chlorophytum comosum* (Sp).

As shown in (Fig. 16) Hierarchical clustering analysis (HCA) provided insightful grouping patterns among the four plant species based on their ability to remove various indoor air pollutants. In the case of total volatile organic compounds (TVOCs), the dendrogram revealed three primary clusters. *Epipremnum aureum* (Ja) was closely grouped with the blank sample, indicating its relatively low removal efficiency. In contrast, the other three species *Syngonium podophyllum* (Sy), *Chlorophytum comosum* (Sp), and *Cordyline fruticosa* (Co) were grouped together in a separate cluster, reflecting their higher VOC removal capacities.

**Fig. 17:**
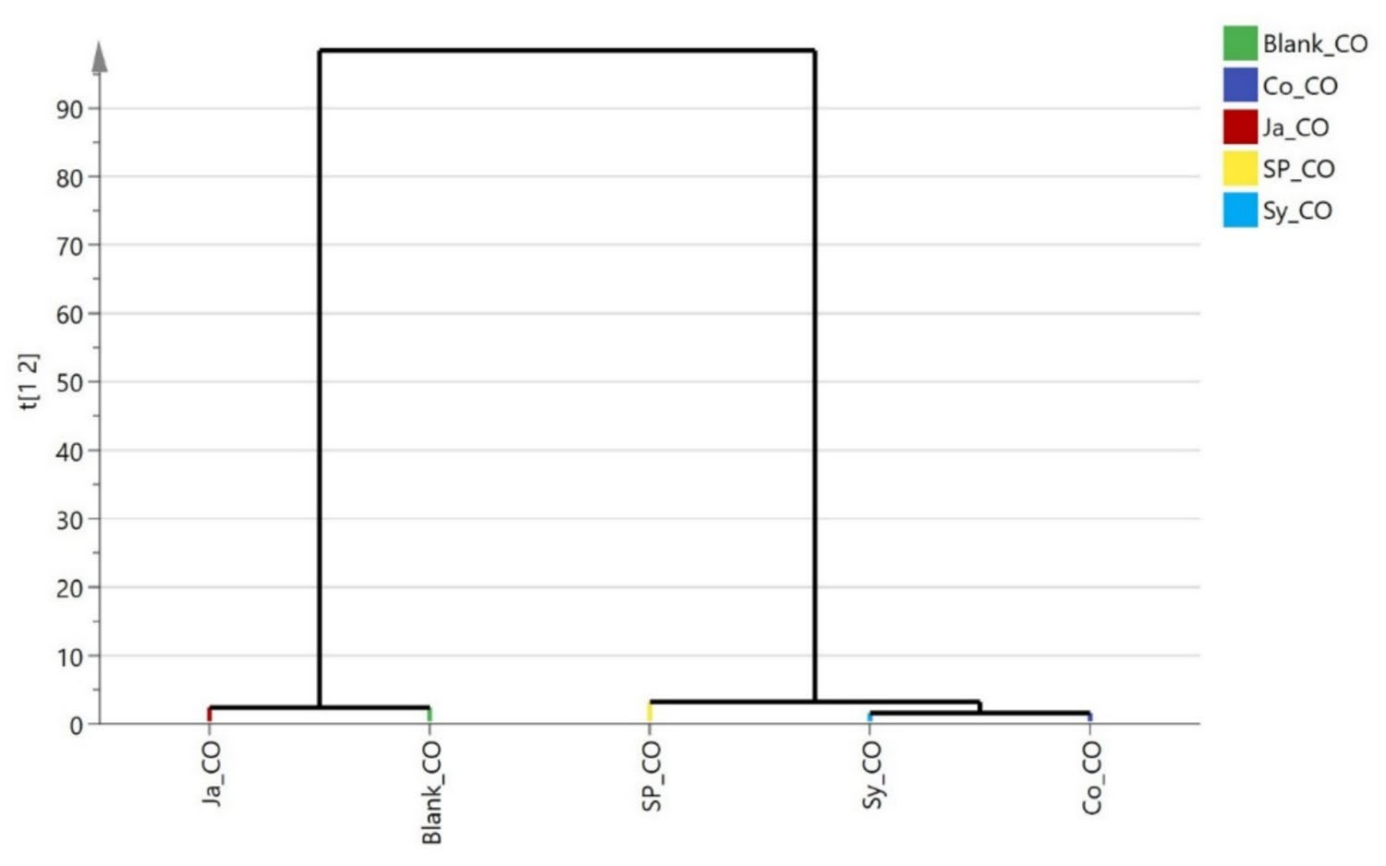
Hierarchical clustering (HCA) plot of the rate of removal of CO by the studied plants *Cordyline fruticose* (Co), *Epipremnum aureum* (Ja), *Syngonium podophyllum* (Sy), *Chlorophytum comosum* (Sp).

The dendrogram (Fig. 17) for carbon monoxide (CO) removal, classified the samples into two main clusters. The first cluster included the blank sample and *Epipremnum aureum* (Ja), suggesting low CO removal potential. The second cluster was subdivided into two sub-clusters: one containing only *Chlorophytum comosum* (Sp), and the other including both *Syngonium podophyllum* (Sy) and *Cordyline fruticosa* (Co), indicating that Sy and Co exhibited comparable and relatively higher CO removal efficiencies, followed by Sp.

**Fig. 18:**
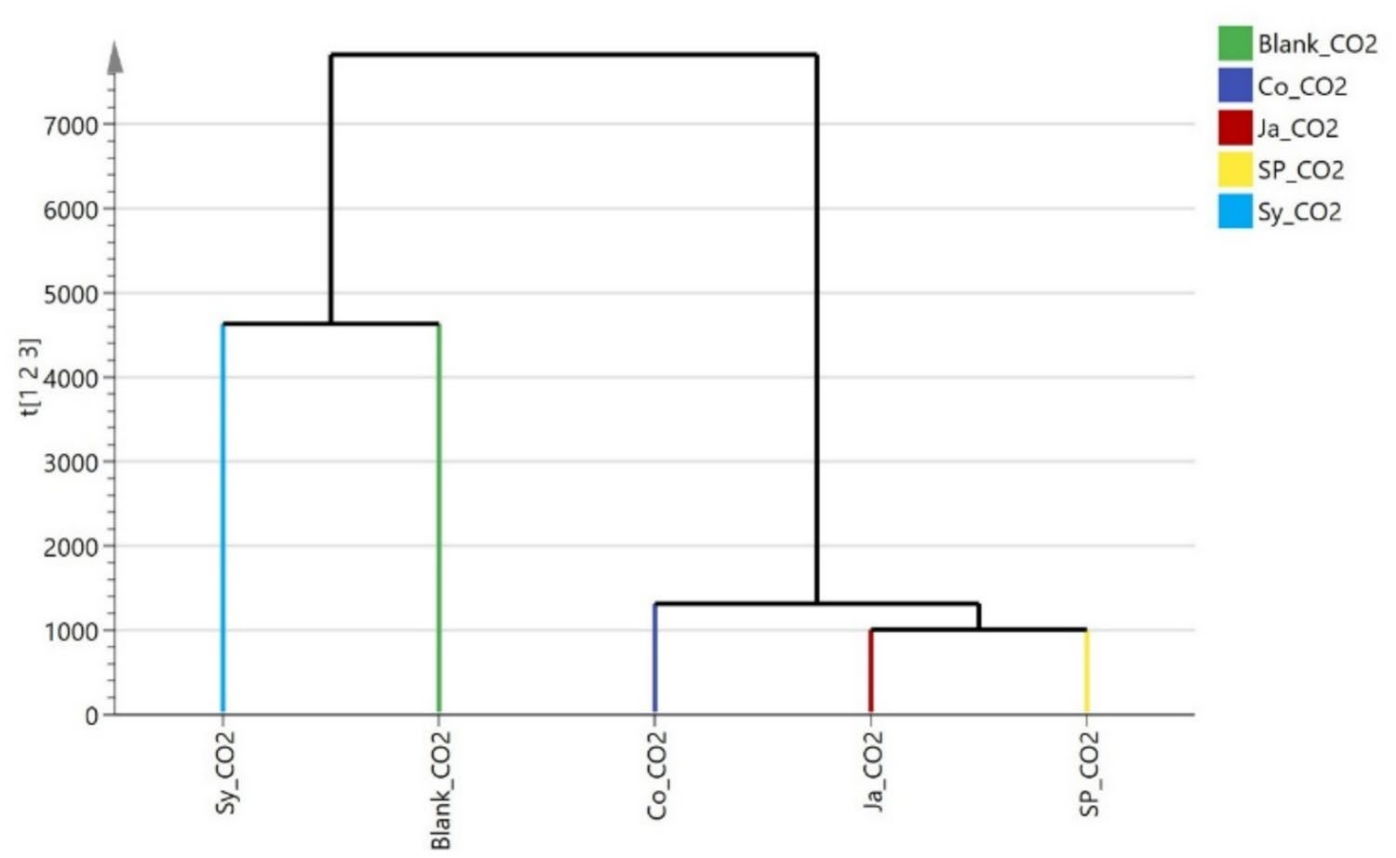
Hierarchical clustering (HCA plot) of the rate of removal of CO_2_ by the studied plants (*Cordyline fruticose* (Co)*, Epipremnum aureum* (Ja)*, Syngonium podophyllum* (Sy), *Chlorophytum comosum* (Sp).

The HCA dendrogram (Fig. 18) again showed two distinct clusters when examining CO₂ removal, the first cluster included *Syngonium podophyllum* (Sy) and the blank sample, suggesting the least effectiveness in removing CO₂. The second cluster was further divided into two subgroups: one containing *Epipremnum aureum* (Ja) and *Chlorophytum comosum* (Sp), and the other containing only *Cordyline fruticosa* (Co). This grouping indicates higher CO₂ removal efficiency by Ja and Sp, with Co being the most effective.

**Fig. 19:**
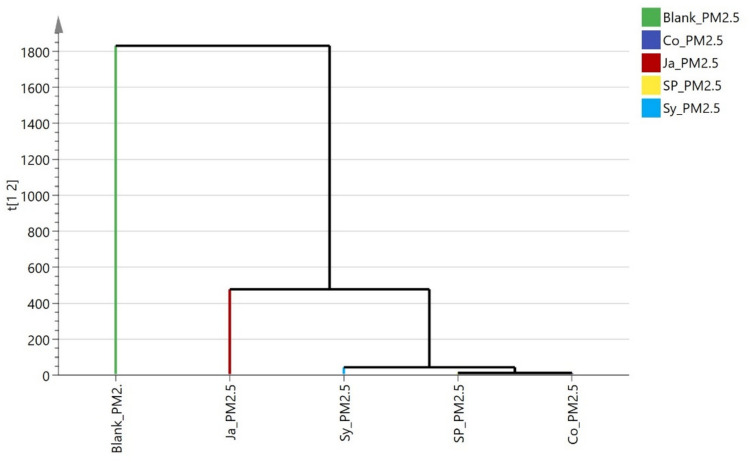
Hierarchical clustering (HCA) plot of the rate of removal of PM_2.5_ by the studied plants (*Cordyline fruticose* (Co)*, Epipremnum aureum* (Jp)*, Syngonium podophyllum* (Sy), *Chlorophytum comosum* (Sp).

**Fig. 20:**
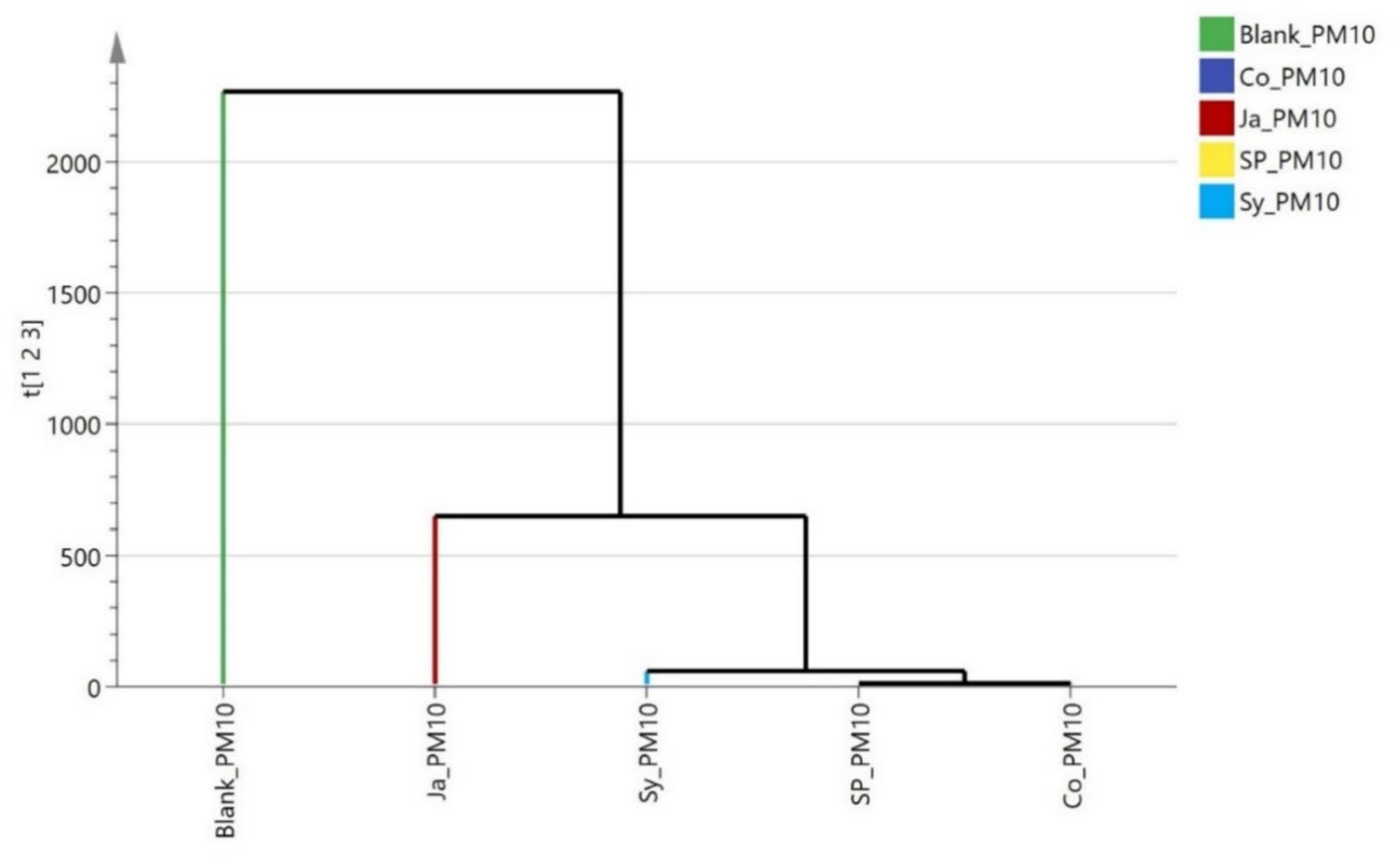
hierarchical clustering (HCA) plot of the rate of removal of PM10 by the studied plants (Cordyline fruticose (Co), Epipremnum aureum (Jp), Syngonium podophyllum (Sy), Chlorophytum comosum (Sp)..

**Fig. 21:**
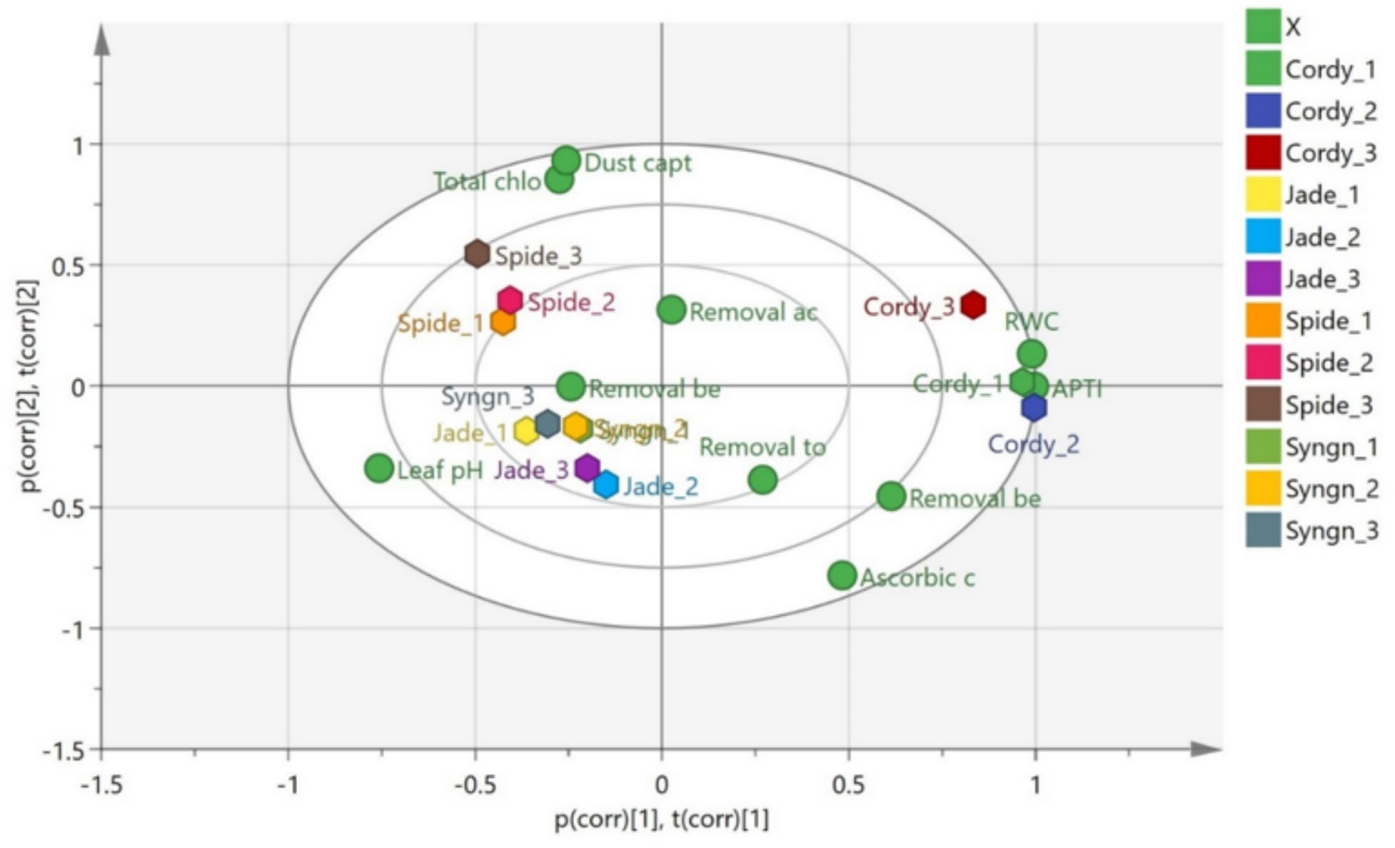
OPLS-DA score plot derived from modelling morphological and physiological indices of the studied plants. P (cor) [1] of the variables of the discriminating component of the OPLS-DA model. Cut-off values of P<0.05 were used.

The dendrograms (Figs. 19, 20) regarding particulate matter (PM2.5 and PM10), showed similar clustering patterns. *Epipremnum aureum* (Ja) formed a separate subcluster, while *Syngonium podophyllum* (Sy), *Chlorophytum comosum* (Sp), and *Cordyline fruticosa* (Co) were grouped together, reflecting their superior performance in reducing particulate matter compared to Ja. These HCA results consistently highlight *Epipremnum aureum* (Ja) as the least efficient species in air pollutant removal, while *Cordyline fruticosa* (Co), *Chlorophytum comosum* (Sp), and *Syngonium podophyllum* (Sy) demonstrated more robust phytoremediation capabilities across multiple pollutants.

(Fig. 21) represents the biplot displaying a supervised multivariate like the orthogonal projection to latent structures-discriminant analysis (OPLS-DA) of the physiological and morphological indices of the plants under study. (OPLS-DA) technique was carried out further to discriminate the plants’ efficiency based on their morphological and physiological indices of the plants. OPLS-DA model showed the separation of the four plants, with R2X = 0.907 and Q2 = 0.689 indicating good fitness and predictability. OPLS-DA score plot (Fig. 22) demonstrated 76.2% of the total sample variance revealed that the efficient removal of benzene the relative water content and the best APTI were correlated the most to **Co** plants and the best removal efficiency of benzene was observed in Jp and Sy which also possessed the most prominent ascorbic acid content. The dust capturing potential and the total chlorophyll were more correlated to the phytoremediation potential of Sp plants.

Based on their morphological and physiological indicators, the plant species are grouped into separate groups as shown by the biplot. Differentiating factors for *Cordyline* include its greater RWC and VOC removal efficiency; for *Chlorophytum comosum*, it’s the pH of its leaves; and for *Syngonium*, its dust capture and chlorophyll content. This analysis aids in comprehending how the morphological and physiological characteristics of these plants contribute to their efficacy in purifying the air and providing other environmental advantages.

#### Implementing applications

Research studies and EPA estimates indicate the number of plants per unit area required to filter indoor air effectively; larger plants with higher leaf surface areas yield better results. In small areas like pharmaceutical labs, plant-based bio-filter indoor air purifiers offer a practical, cost-effective, and eco-friendly means of improving IAQ. Overall Impact on IAQ: By combining ventilation systems with plant-based bio-filters, significant improvements in IAQ can be attained, improving people’s health and productivity in indoor environments. These findings corroborate current research and highlight the potted plants’ potential as effective indoor air purifiers.

#### Novelty of the study

This study presents a novel and integrated approach to indoor air phytoremediation by systematically evaluating the performance of four ornamental plant species across real-life indoor environments, as operational pharmaceutical laboratories. Unlike previous studies that rely on a single performance indicator, this work introduces a multi-metric assessment framework, combining removal efficiency (RE%), normalization by leaf area, and total absorbed VOC mass to provide a more comprehensive evaluation of plant performance. Furthermore, the study advances current knowledge by exploring the practical implementation of plant-based biofilter systems (PBBFs) under realistic laboratory conditions, bridging the gap between experimental research and real-world applications. The inclusion of multivariate analysis and Air Pollution Tolerance Index (APTI) evaluation further strengthens the scientific contribution by enabling a deeper understanding of species-specific responses and selection criteria for sustainable indoor air quality management.

## Conclusion

This study highlights the potential of using ornamental plants as an effective and sustainable solution for improving indoor air quality in pharmaceutical laboratories. Among the tested species, *Cordyline fruticosa* and *Syngonium podophyllum* consistently demonstrated superior performance across multiple evaluation criteria. The high Air Pollution Tolerance Index (APTI) values observed particularly for *Cordyline fruticosa* were supported by elevated levels of total chlorophyll, relative water content (RWC), leaf extract pH, and ascorbic acid, indicating strong physiological resilience under VOCs exposure. Additionally, key morphological traits such as high stomatal density and abundant cuticle wax contributed significantly to pollutant capture and absorption. Multivariate chemometric analyses, including PCA and OPLS-DA, further confirmed the strong correlation between these physiological traits and VOCs removal efficiency. The clustering patterns and distinct component separations demonstrate potential of the selection of these species as optimal biofilters. Taken together, the findings not only underscore the importance of selecting plant species based on integrated physiological and biochemical criteria but also demonstrate the value of chemometric tools in guiding effective phytoremediation strategies for indoor air quality management.

## Recommendations

Integrate a variety of plant species to target a broad spectrum of VOCs. Position plants strategically maximize their exposure to polluted air. Also maintain plant health to ensure consistent VOCs absorption rates. Furthermore, further studies should be conducted to investigate the long-term effects and optimal conditions for the use of different plant species such as PBBs. Integrate high-performing ornamental plants such as *Cordyline fruticosa* and *Syngonium podophyllum* into indoor environments, especially in pharmaceutical laboratories where VOCs exposure is a concern. These species can be strategically arranged in green walls or plant-based biofilter systems to naturally reduce harmful pollutants. Future efforts should focus on long-term monitoring, incorporating multiple plant species, and optimizing growing conditions, including light and substrate composition, to maximize phytoremediation efficiency. Additionally, using physiological indicators like APTI, stomatal density, and wax content as screening tools can help identify other resilient plant candidates for sustainable indoor air quality improvement.

## Limitations of the study

While this study provides encouraging evidence on the use of ornamental plants as natural air purifiers in pharmaceutical labs, it’s important to recognize a few limitations. Much of the testing was done in controlled environments, which doesn’t fully reflect the day-to-day changes and interactions found in real lab settings. The focus was on only four VOCs, leaving out other common pollutants that might behave differently. Also, each test used just one plant per setup, so the collective effects of mixed-species green walls weren’t explored. The study period was relatively short, meaning long-term plant performance and maintenance needs remain unclear. Additionally, we didn’t assess the role of root-zone microbes or growing media, even though they can significantly boost pollutant breakdown. Finally, while we evaluated health risks for benzene and toluene, a broader health impact assessment covering more VOCs would provide an even clearer picture of the benefits. These insights will help guide future research in making indoor plant-based air cleaning systems even more effective and practical.

## Data Availability

The datasets generated and analyzed during this study, are available from the corresponding aut hor upon reasonable request.
